# A qualitative investigation of healthcare professionals’ viewpoints of the healthcare process of persons with a serious mental illness in prisons with a traditional model for mental health care provision in Spain

**DOI:** 10.1192/j.eurpsy.2023.405

**Published:** 2023-07-19

**Authors:** A. Calcedo-Barba, S. Paz Ruiz, V. Estévez Closas, Á. López López, L. F. Barrios, J. Antón Basanta

**Affiliations:** 1 Sociedad Española de Psiquiatría Legal, Madrid; 2 SmartWorking4U SLU; 3 SmartWorking4U, Benicassim; 4 Hospital Psiquiátrico Penitenciario, Fontcalent; 5 Universidad de Alicante, Alicante; 6 Sociedad Española de Sanidad Penitenciaria, Barcelona, Spain

## Abstract

**Introduction:**

Healthcare delivery in prisons depends on the national Ministry of Interior in 14 of 17 autonomous regions in Spain. A traditional model for health and mental health care provision prevails.

**Objectives:**

To increase understanding of the mental health care process of imprisoned persons with a serious mental illness (SMI) in Spanish prisons with a traditional model of health care provision.

**Methods:**

10 healthcare professionals (6 physicians, 3 nurses, 1 pharmacist) working in small (<450 imprisoners), middle size (450-1,000) and big (>1,000) prisons took part in 3 online focus groups between 31^st^ May and 2^nd^ June 2022. The moderator used open-ended questions to research into the healthcare process (diagnosis, treatment, follow up, prevention) of imprisoners with SMI. Focus groups lasted 2 hours, and were audiotape recorded and transcribed. Transcripts were analysed applying constant comparative method and theoretical saturation.

**Results:**

Mental healthcare provision varies across prisons, but commonalities exist. Healthcare professionals reported that about 60% of SMI are diagnosed by the correctional general practice physician (GP) at incarceration. Severe cases are assessed by an external psychiatrist. Once a week (average) the psychiatrist visits the prison to either confirm diagnoses or adjust treatments. One third of imprisoners who would benefit from a psychiatric assessment has it. Follow up occurs in the prison infirmary for close supervision. If addiction concurs, referral to therapeutic modules happen. Polypharmacy and overmedication are common. Simplification of therapies and slow-release injectable formulations of antipsychotics are desirable. Everyday mental health care and rehabilitation take place throughout a specific, little equipped, psycho-social support programme implemented in most prisons but restricted to the most disabled SMI persons. Acute psychiatric episodes occur due to treatment interruptions or deviations and are managed by the correctional GP. Hospital referrals are problematic without protocols. Prevention of relapses relies on imprisoners supervision and staff observation. Healthcare records are only available to healthcare professionals working in prisons. Outside prisons, continued care needs of mental health and social support in the community. Due to healthcare services modest readiness to respond to needs and poor social networks, SMI persons are prone to relapse and recidivism.

**Conclusions:**

Focus groups found that working in isolation from the public healthcare system, shortage of psychiatrists, poorly implemented therapeutic and rehabilitation programmes, and lack of mental health and social care services in the community negatively affect the care of imprisoners with SMI in Spain.

**Disclosure of Interest:**

None Declared

